# Residents of the towns in which the Fukushima Daiichi nuclear station is located express more worries about reputational damage than about the discharge of treated water itself

**DOI:** 10.1093/jrr/rraf003

**Published:** 2025-02-16

**Authors:** Mengjie Liu, Hitomi Matsunaga, Makiko Orita, Yuya Kashiwazaki, Xu Xiao, Noboru Takamura

**Affiliations:** Department of Global Health, Medicine and Welfare, Atomic Bomb Disease Institute, Nagasaki University Graduate School of Biomedical Sciences, 1-12-4 Sakamoto, Nagasaki 852-8523, Japan; Department of Global Health, Medicine and Welfare, Atomic Bomb Disease Institute, Nagasaki University Graduate School of Biomedical Sciences, 1-12-4 Sakamoto, Nagasaki 852-8523, Japan; Department of Global Health, Medicine and Welfare, Atomic Bomb Disease Institute, Nagasaki University Graduate School of Biomedical Sciences, 1-12-4 Sakamoto, Nagasaki 852-8523, Japan; Department of Global Health, Medicine and Welfare, Atomic Bomb Disease Institute, Nagasaki University Graduate School of Biomedical Sciences, 1-12-4 Sakamoto, Nagasaki 852-8523, Japan; Department of Global Health, Medicine and Welfare, Atomic Bomb Disease Institute, Nagasaki University Graduate School of Biomedical Sciences, 1-12-4 Sakamoto, Nagasaki 852-8523, Japan; Department of Global Health, Medicine and Welfare, Atomic Bomb Disease Institute, Nagasaki University Graduate School of Biomedical Sciences, 1-12-4 Sakamoto, Nagasaki 852-8523, Japan

To the Editor:

In August 2023, the regular discharge of treated water (DTW) from the Tokyo Electric Power Company’s Fukushima Daiichi Nuclear Power Station (FDNPS) into the Pacific Ocean began. The Japanese government and the Tokyo Electric Power Company have conducted a series of dialogues and informed the public about this process, to enhance their understanding of the discharge protocol [1]. Furthermore, they have undertaken careful preparations, including the accumulation of scientific data regarding the environmental and human health implications of the discharge, and they have sought water-handling recommendations from the IAEA [2]. Nevertheless, public concerns persist [3].

The DTW site is located at the FDNPS, in Okuma and Futaba Towns. Immediately after the FDNPS accident, all residents were ordered to evacuate and forbidden to enter the towns without permission from the government. Although the evacuation orders for Okuma and Futaba Towns were partially lifted almost a decade later, in 2020 and 2022, respectively, the return rates for both towns have been very low, which is attributed to the varying consequences of the prolonged evacuation [4]. Moreover, residents of the towns have expressed deep-rooted concerns about radiation exposure and its health effects, including DTW [5–7].

Between November and December 2023, ~3 months after the DTW’s initiation, a questionnaire survey was conducted of all residents of Okuma and Futaba Towns aged 18 years and older who had a residence card at the time of the survey. The questionnaire was distributed and enclosed in public magazines to ~4900 households in Okuma and 2600 households in Futaba. After excluding incomplete responses, 549 responses from Okuma and 419 from Futaba were deemed valid. The study protocol was approved by the Ethics Committee of the Nagasaki University Graduate School of Biomedical Sciences (Approval No. 23081805).

The survey results showed that 40.3% (*n* = 390) of respondents expressed concerns about DTW itself, whereas 68.2% (*n* = 660) were worried about reputational damage due to DTW (Table 1). Of all the reputational damage, the biggest worry was the impact on the fishing industry 76.6% (*n* = 783) by the multiple answers. Subsequent worries were related to its impact on commerce (*n* = 330, 32.3%), tourism (*n* = 298, 29.2%), their livelihood (*n* = 74, 7.2%), and other factors (*n* = 106, 10.4%). A regression model was calculated separately for current place of residence (Model 1) and respondents’ intention to return (Model 2), because collinearity was confirmed (Table 2). The regression analysis results showed that, compared to males, females were more concerned about DTW (Model 1, odds ratio [OR]: 0.59, 95% confidence interval [Cl]: 0.45–0.76, *P* < 0.001; Model 2, OR: 0.59, CI: 0.46–0.77, *P* < 0.001). Furthermore, compared to those aged <65 years, those aged ≥65 years were more worried about reputational damage caused by DTW (Model 1, OR: 1.40, Cl: 1.05–1.87, *P* < 0.05; Model 2, OR: 1.40, CI: 1.05–1.87, *P* < 0.05). Notably, those who have not returned to the area were more concerned about DTW than those who have returned or who were living there (Model 2, OR: 2.46, CI: 1.05–5.73, *P* < 0.05). This result was similar to a previous study that showed people who were female or who had decided not to return tended to be more anxious about radiation exposure and its health effects following the nuclear accident [5, 6]. We found no statistical differences in worries about reputational damage depending on the respondents’ current residential area or intention to return. In other words, the residents of the towns located near the FDNPS worried about reputational damage, regardless of whether they have decided to return or not, or where they live at present.

**Table 1:** Residents’ concerns about DTW-related reputational damage

	Characteristic	Overall	Concerned about DTW		Concerns about reputational damage caused by DTW	
		n (%)	n (%)		n (%)	
		968 (100)	390 (40.3)	*P*-value	660 (68.2)	*P*-value
Sex	Male Female	536 (55.4) 432 (44.6)	185 (47.4) 205 (52.6)	<0.01	357 (54.1) 303 (45.9)	0.24
Age (years)	< 65 ≥ 65	355 (36.7) 613 (63.3)	144 (36.9) 246 (63.1)	0.89	258 (39.1) 402 (60.9)	0.02
Place of residence	Hamadori area; Pacific coast side Inside Fukushima; not including Hamadori Outside Fukushima	487 (50.3) 207 (21.4) 274 (28.3)	191 (49.0) 82 (21.0) 117 (30.0)	0.63	325 (49.2) 140 (21.2) 195 (29.5)	0.44
Intention to return	Already returned or living in area Want to return Undecided Do not want to return	34 (3.5) 126 (13.0) 253 (26.1) 555 (57.3)	7 (1.8) 53(13.6) 107 (27.4) 223 (57.2)	0.11	18 (2.7) 86 (13.0) 183 (27.7) 373 (56.5)	0.12

*Note.* Response: yes, Chi-square test, DTW: discharge of treated water

**Table 2:** Independent factors associated with anxiety and reputational damage related to DTW

		Concerned about DTW		Concerned about reputational damage due to DTW	
		Model 1 OR (95%Cl)	Model 2 OR (95% Cl)	Model 1 OR (95% Cl)	Model 2 OR (95% Cl)
Sex	Male/Female	0.59** (0.45–0.76)	0.59** (0.46–0.77)	0.87 (0.66–1.14)	0.88 (0.67–1.15)
Age (years)	< 65/≥ 65	0.99 (0.76–1.31)	1.01 (0.77–1.32)	1.40* (1.05–1.87)	1.40* (1.05–1.87)
Place of residence	Other/ Hamadori area; Pacific coast	1.06 (0.82–1.38)		1.16 (0.88–1.52)	
Intention to return	Other/ Already returned or living in area		2.46* (1.05–5.73)		1.97 (0.98–3.93)

*Note.* Reference; yes, logistic regression analysis, DTW: discharge of treated water, OR: odds ratio, Cl: confidence interval, *P* < 0.05*, *P* < 0.01**

Compared with the timing of the initial DTW in 2023, the frequency of media coverage of DTW has decreased significantly over time. Impressively, the residents of the towns where the FDNPS is located clarified their decreasing perception of the risks to their health and genetic effects because of the DTW after the process started [7]. However, laypeople remain concerned, and these concerns might generate harmful rumors and misunderstandings about the DTW process, despite the inclusion of incredibly low levels of radionuclides in treated water [8]. Residents of the towns where the FDNPS is located were more worried about reputational damage than about DTW itself, including the health and environmental effects. This finding highlights the negative impact on society caused by insufficient or misleading information and the necessity of evaluating both domestic and international reactions to social and psychological consequences among the general population. It would be suggested that an important comparative analysis between the severity of social and psychological reactions in the population living near the FDNPS and the population that is not directly affected by these events but receives information about them only from the mass media.

It is essential to develop information to mitigate reputational damage related to DTW and develop human resources, both domestically and internationally, who can effectively communicate and disseminate information based on scientific evidence. In particular, both the health effects and how essential the DTW process is need to be better understood among those in medicine, the government, related fields, and the general public. Rumors are not only an issue of human rights but also cause real harm that generates economic losses [9]. Japan has experienced trade restrictions on seafood as a result of DTW, thus the government has a responsibility to restore the international trust and brand power of the Japanese seafood industry. Addressing the reputational damage of DTW will not only mitigate the anxiety experienced by victims of nuclear accidents but could also be an important means of preventing the deterioration of social trust among the global community.

## PRESENTATION AT A CONFERENCE

No.
